# Leptin receptor signaling is required for high-fat diet-induced atrophic gastritis in mice

**DOI:** 10.1186/s12986-016-0066-1

**Published:** 2016-02-02

**Authors:** Kyoko Inagaki-Ohara, Shiki Okamoto, Kazuyo Takagi, Kumiko Saito, Seiya Arita, Lijun Tang, Tetsuji Hori, Hiroaki Kataoka, Satoshi Matsumoto, Yasuhiko Minokoshi

**Affiliations:** Research Institute, National Center for Global Health and Medicine (NCGM), 1-21-1, Toyama Shinjuku, Tokyo, 162-0052 Japan; Division of Endocrinology and Metabolism, Department of Developmental Physiology, National Institute for Physiological Sciences (NIPS), 38 Nishigonaka Myodaiji, Okazaki, Aichi 444-8585 Japan; Division of Host Defense, Department of Life Sciences, Faculty of Life and Environmental Sciences, Prefectural University of Hiroshima, 562 Nanatsuka, Shobara, Hiroshima 727-0023 Japan; Yakult Central Institute for Microbiological Research, 5-11 Izumi, Kunitachi, Tokyo, 186-8650 Japan; Section of Oncopathology and Regenerative Biology, Department of Pathology, Faculty of Medicine, University of Miyazaki, 5200 Kihara, Kiyotake, Miyazaki, 889-1692 Japan

**Keywords:** Leptin, Atrophic gastritis, High-fat diet, Obesity

## Abstract

**Background:**

Obesity increases the risk for malignancies in various tissues including the stomach. Atrophic gastritis with precancerous lesions is an obesity-associated disease; however, the mechanisms that underlie the development of obesity-associated atrophic gastritis are unknown. Leptin is a hormone derived from stomach as well as adipose tissue and gastric leptin is involved in the development of gastric cancer. The aim of the current study is to investigate the involvement of leptin receptor signaling in the development of atrophic gastritis during diet-induced obesity.

**Methods:**

Male C57BL/6, *ob/ob* and *db/db* mice were fed a high-fat diet (HFD) or a control diet (CD) from 1 week to 5 months. Pathological changes of the gastric mucosa and the expression of molecules associated with atrophic gastritis were evaluated in these mice.

**Results:**

HFD feeding induced gastric mucosal hyperplasia with increased gastric leptin expression. Mucosal hyperplasia was accompanied by a higher frequency of Ki67-positive proliferating cells and atrophy of the gastric glands in the presence of inflammation, which increased following HFD feeding. Activation of ObR signaling-associated molecules such as ObR, STAT3, Akt, and ERK was detected in the gastric mucosa of mice fed the HFD for 1 week. The morphological alterations associated with gastric mucosal atrophy and the expression of Muc2 and Cdx2 resemble those associated with human intestinal metaplasia. In contrast to wild-type mice, leptin-deficient *ob/ob* mice and leptin receptor-mutated *db/db* mice did not show increased Cdx2 expression in response to HFD feeding.

**Conclusion:**

Together, these results suggest that activation of the leptin signaling pathway in the stomach is required to develop obesity-associated atrophic gastritis.

**Electronic supplementary material:**

The online version of this article (doi:10.1186/s12986-016-0066-1) contains supplementary material, which is available to authorized users.

## Background

Gastric carcinoma (GC) typically arises on a background of atrophic gastritis, intestinal metaplasia, and dysplasia of gastric mucosa, and is the second leading cause of cancer-related deaths worldwide [1]. Obesity augments the risk of a higher prevalence of gastritis [2, 3], atrophic gastritis [4–6], and gastric cardia adenocarcinoma [7–9]. Infection with *Helicobacter pylori*, a bacterium that infects humans and colonizes the stomach, is the predominant cause of precancerous lesions in the mucosal lining of the stomach [10]. Although *H. pylori* infection is not confined to morbidly obese patients, obesity increases the prevalence of chronic gastritis and GC [2]. Furthermore, obesity is not only a risk factor for certain tumors but is also associated with an increased mortality rate [11]. Thus, obesity potentially affects the development of gastritis into gastric tumorigenesis. Therefore, it is imperative to identify signaling molecules associated with both obesity and precancerous lesions to aid in the management of high-risk individuals.

Leptin, a product of the obese (*ob*) gene, is primarily produced by adipocytes and acts on its receptor (ObR) in the hypothalamus to suppress food intake and increase energy expenditure [12]. ObR belongs to the class I cytokine receptor family, and its structure is highly homologous to that of gp130, the common signal-transducing receptor for the interleukin-6 (IL-6) family of cytokines [13]. Of the six alternate splice variants of ObR, only the long isoform, ObRb, transduces a signal cascade that activates downstream Janus kinase 2 and signal transducer and activator of transcription 3 (JAK2-STAT3), phosphoinositide 3-kinase (PI3K), and extracellular signal-regulated kinase 1/2 (ERK1/2) [14]. In addition to its role in energy homeostasis, leptin exerts pleiotropic effects on angiogenesis, hematopoiesis, and immunity as well [14]. Leptin and ObR are also expressed in various tissues including the gastrointestinal tract [15]. Additionally, the stomach can spontaneously express leptin and ObR, leading to the augmentation of leptin receptor signaling in the stomach during GC development [16–18]. We have previously demonstrated the significance of leptin signaling in the stomach and its role in the development of intestinal-type gastric tumor using a murine model [19]. Dysfunction of central sympathetic regulation of leptin signaling promotes leptin resistance. Despite high levels of circulating plasma leptin, obese individuals do not respond to its appetite-suppressing effects, indicating their leptin resistance [20]. Because leptin is crucial to the development of gastrointestinal malignancies and provides a link between obesity and tumorigenicity [17], a better understanding of the dysregulation of gastric leptin signaling and its role in obesity-induced gastric pathology is necessary.

## Methods

### Animals and diets

Male C57BL/6J (wild-type: WT), *ob/ob*, and *db/db* mice (CLEA Japan, Tokyo, Japan) were studied at 7 weeks of age. The animals were housed individually in plastic cages at 24 °C ± 1 °C with lights on from 0600 to 1800 h. The mice were provided with either a control-diet (CD, 10 % of calories from fat, D12450J) or a high-fat diet (HFD, 60 % of calories from fat, D12492) (Research Diets Inc., New Brunswick, NJ) and water *ad libitum*. The ethics committee for animal experiments of the National Institute for Physiological Sciences approved all animal experiments.

### Histopathological analysis of the gastric mucosa

Paraffin-embedded gastric sections of 10 % formalin-fixed tissues were obtained from the HFD- and CD-fed mice and were stained with hematoxylin and eosin (H&E), and assessed for alterations in the gastric mucosa. The assessment of mucosal alterations in the stomach was based on a summation of scores for hyperplasia (0, non-substantial alteration; 1, low; 2, moderate; 3, high), cell infiltration (0, non-substantial alteration; 1, low; 2, moderate; 3, high), loss of gastric glandular cells (0, non-substantial alteration; 4, low; 5, moderate; 6, high), Alcian blue staining (0, non-substantial alteration; 4, focal; 5, diffuse; 6, very strong diffuse), and dysplasia (0, non-substantial alteration; 7, low). Each criterion was independently blind-scored by two individuals using criteria that were previously defined [19].

### Intragastric pH measurements

Gastric pH was measured according to a published method [21]. In brief, mice were sacrificed after anesthetization by carbon dioxide inhalation. Following stomach removal, the gastric lumen was removed and washed with 0.5 ml saline (150 mM, pH 7.0), and the pH of the collected gastric fluid was measured using a pH meter (Mettler, Toledo, OH).

### Immunohistochemical analysis

Paraffin-embedded sections of 10 % formalin-fixed tissues were stained either with H&E or with periodic-acid Schiff (PAS) and Alcian blue. For antigen retrieval, deparaffinized and rehydrated specimens were treated with 3 % hydrogen peroxide in methanol to block endogenous peroxidase activity and then were heated in a microwave using a Retrievagen A kit (BD Biosciences, San Jose, CA), followed by overnight incubation with primary antibodies (Abs) at 4 °C as listed in Additional file 1: Table S1. Subsequently, the slides were stained with a biotinylated anti-rabbit IgG or anti-goat IgG Ab and streptavidin-labeled peroxidase using a Histofine SAB-PO kit (Nichirei Biosciences Inc., Tokyo, Japan) and developed using 3, 3′-diaminobenzidine (DAB) solution (ImmPact^TM^ DAB, Vector Laboratories, Burlingame, CA) according to the manufacturer’s protocol, followed by hematoxylin counterstaining. For immunofluorescence staining, the slides were incubated with the primary Abs (Additional file 1: Table S1) and then reacted with Alexa 488-conjugated rabbit or mouse IgG Ab or Alexa 556-conjugated goat IgG Ab, as appropriate. The stained slides were mounted using ProLong Gold Antifade reagent with 4′,6-diamidino-2-phenylindole (DAPI) (Life Technologies, Carlsbad, CA) for detection using a fluorescence microscope (Olympus, Tokyo, Japan).

### Western blot analysis

Gastric epithelial cells were isolated and prepared according to a modification of a previously published method [22]. Dissected small segments of the stomach were agitated at room temperature for 10 min in a Hank's balanced salt solution (HBSS) (Thermo Fisher Scientific Inc., Waltham, MA) medium containing 1 mM DTT. After removal of the supernatant, the tissues were stirred at 37 °C for 10 min in HBSS containing 10 mM EDTA. After removal of the supernatant, the tissue suspension was passed through a nylon mesh to remove debris and centrifuged through a 25/40 % discontinuous Percoll (Sigma-Aldrich, St. Louis, MO) gradient at 600 × *g* at 20 °C for 20 min. The cells collected from the interface of 25/40 % were the epithelial cells. Lysates were prepared from tissues and cells and analyzed by western blotting, according to a previously published method [23]. The Abs used in western blotting are summarized in Additional file 1: Table S1.

### Laser-capture microdissection

The above-described paraffin-embedded gastric tissues were cut into 6-μm-thick sections and mounted onto membrane slides (MembraneSlide 1.0 PEN, Carl Zeiss Microscopy, LLC, Thornwood, NY). Paraffin was removed by rinsing the sections with xylene, after which the sections were immersed in a series of 100 % to 70 % ethanol baths and air-dried. Mucosal sections of gastric epithelia were cut and collected onto AdhesiveCaps (PALM, Microlaser Technologies, Bernried, Germany) by a laser-capture microdissection (LMD) system (PALM MB-III, Microlaser Technologies).

### Quantitative reverse transcription-polymerase chain reaction (qRT-PCR)

Total RNA from the LMD samples and from murine gastric mucosa was extracted using AllPrep FFPE DNA/RNA and RNeasy Mini kits (Qiagen, Valencia, CA), respectively, according to the manufacturer’s protocols. cDNA was synthesized from approximately 100–200 ng RNA from the LMD sections or 1–2 μg RNA from gastric mucosal cells using the ReverTra Ace^®^ qPCR RT Kit (TOYOBO, Co., Ltd., Osaka, Japan) according to the manufacturer’s protocol. qRT-PCR was carried out using the Power SYBR Green PCR Master Mix (Life Technologies, Carlsbad, CA) with specific primer sets (400 nM at the final concentration, Additional file 2: Table S2) according to the manufacturers’ protocol. Relative changes in gene expression were calculated using the ΔΔCt method, and the 18S rRNA gene was used for normalization.

### Quantitative analysis of immunohistochemical staining

For microscopic measurements, leptin-stained gastric mucosa samples were photographed using a microscope (Olympus), and quantitative analysis was performed using ImageJ software (http://rsb.info.nih.gov/ij/index.html). Mucosal height was measured between the base of the gastric glands and the neck zone.

### Plasma assay

Serum was collected from blood obtained by cardiocentesis under anesthetization and stored at −80 °C. Insulin (Mouse Insulin ELISA kit, Shibayagi, Gunma, Japan), leptin (Leptin ELISA, Millipore, St. Charles, MO), glucose (Glucose CII-test, Wako, Osaka, Japan), and non-esterified fatty acid (NEFA) (NEFA C-test, Wako) levels in the sera were measured according to the manufacturers’ protocols.

### Statistical analysis

The Mann–Whitney *U* test and the Kruskal-Wallis test were used to determine significant differences. A *p*-value of less than 0.05 was considered significant. Statistical analyses were performed using Prism software version 6 (GraphPad, San Diego, CA, USA).

## Results

### HFD-fed mice develop atrophic gastritis

**Fig. 1:**
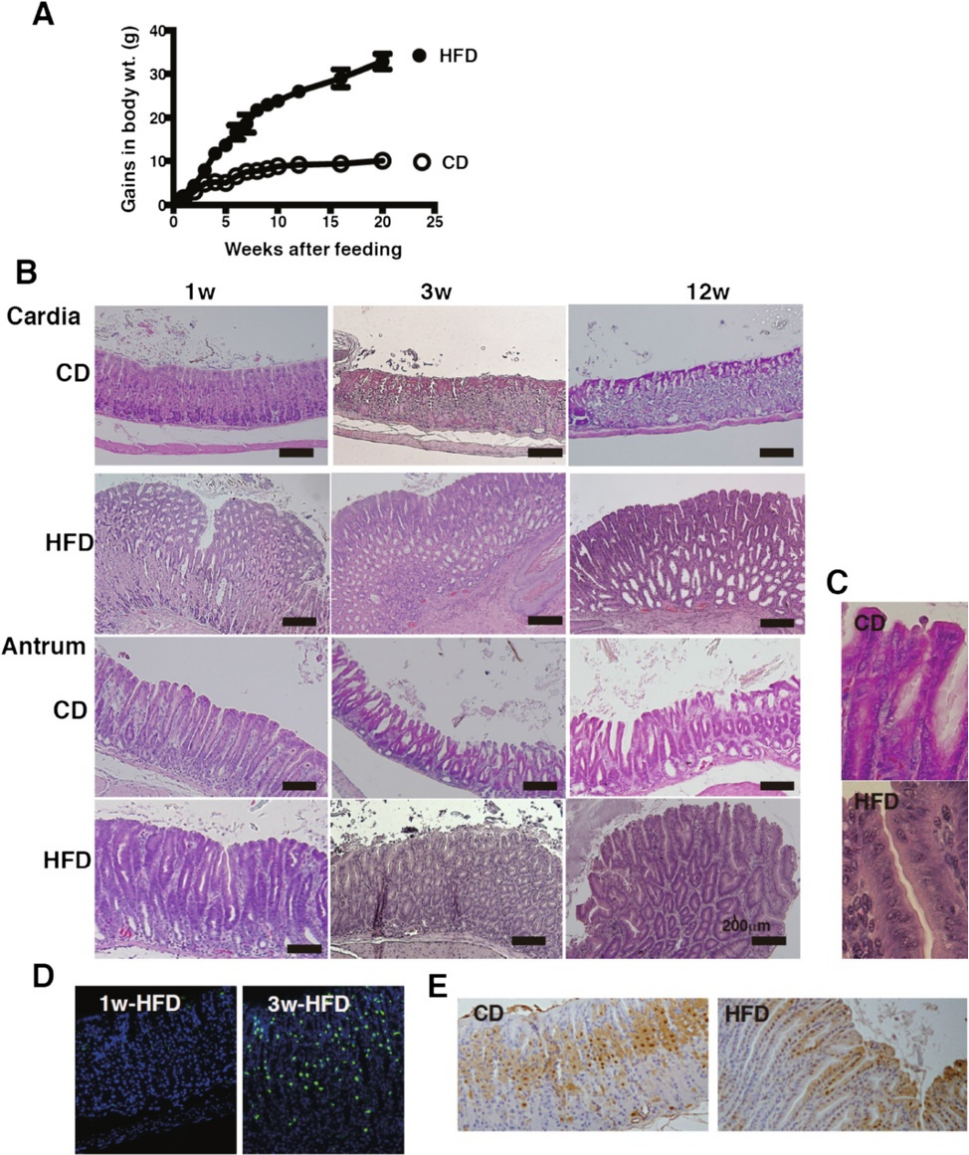
Pathological changes of gastric mucosa owing to HFD-feeding. **a** Alteration of gains in body weights of C57BL/6 J mice fed CD (*n* = 10) or HFD (*n* = 10) during 20 weeks. **b** Representative H&E-sections of the gastric cardia and antrum from mice fed CD or HFD for 1, 3, and 12 weeks. **c** Magnified image of the gastric antrum in mice fed CD and HFD in Fig. 1b at 12 weeks after feeding (magnification, ×400). The cell nucleolus, nuclear hypertrophy, dyspolarity, and pseudostratification were observed. **d** CD45 staining of the gastric mucosa of 1 and 3 week HFD-fed mice. **e** Ki67-staining in the gastric mucosa of mice fed CD or HFD for 3 weeks. 5–10 mice were used in each analysis, and representative data are shown

**Fig. 2:**
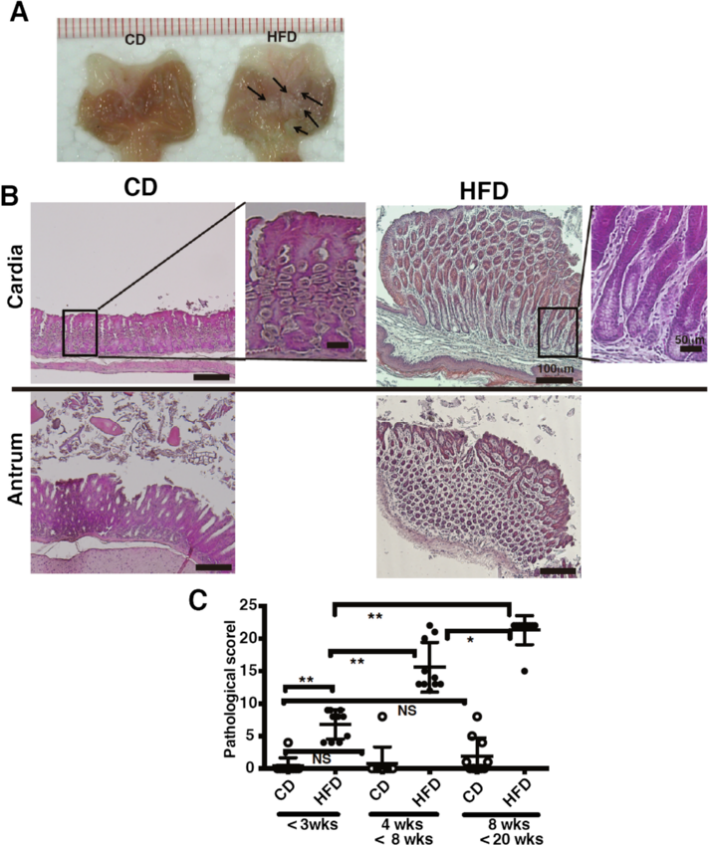
Development of gastric mucosal atrophy in diet-induced obese mice. **a** The gastric lumen was opened along the outer curvature of mice fed CD or HFD for 20 weeks. Arrows indicate the polyp-like lesions in the stomach of HFD-fed mice. **b** Representative H&E-sections of the gastric cardia and antrum from mice fed CD or HFD for 20 weeks. **c** The histological scores from the stomachs of mice fed CD or HFD (<3 weeks, 4–8 weeks, 8–20 weeks of feeding; 10 mice per group) were graded according to the diagnostic criteria described in the Methods. Results were analyzed by the Kruskal-Wallis test, followed by a Dunn’s multiple comparison test. * *p* < 0.05, ** *p* < 0.01, NS; not significant

To determine how diet-induced obesity affects the pathogenesis of gastric mucosa, C57BL/6 mice were fed either HFD (60 % calories from fat) or CD (10 % calories from fat) and the histological changes of the gastric mucosa were examined in a time-dependent manner. Compared to the CD-fed mice, the HFD-fed mice exhibited rapid weight gain at a rate of > 2 g per week during the first 12 weeks. Thereafter, a moderate increase of 1 g per week was observed from 12 to 20 weeks (Fig. 1a). The cardial mucosa showed hyperplasia at 1 week, prior to any significant difference in body weight gain between the CD- and HFD-fed groups (1.5 ± 0.29 in CD *vs*. 1.8 ± 0.4 in HFD, *p* > 0.05) (Fig. 1a and 1b). At 3 weeks, a reduced parietal cell number and morphological alterations of the foveola in the stomach were observed, followed by glandular metaplasia and a complete loss of zymogenic and parietal cells at 12 weeks after the initiation of HFD feeding (Fig. 1b). Although the hyperplastic change of the gastric foveolar epithelium was seen at 1 week in the HFD-fed mice, few CD45^+^ infiltrated cells were present in HFD-fed mice (Fig. 1b and 1d). However, after 3 weeks of feeding, a substantial amount of infiltrated cells were seen to have invaded the interglandular and basal spaces in accordance with the development of hyperplasia (Fig. 1b and 1d). In the antrum, slight mucosal hyperplasia was observed in HFD-fed mice at 1 week after diet initiation. Both the cardia and antrum displayed a replacement of normal glandular cells such as parietal and G cells with atypical and irregular cells after 3 weeks of HFD feeding (Fig. 1b). Furthermore, mildly dysplastic epithelia, with cells showing enlarged nuclei, nuclear pseudostratification, and distinct nucleoli, became apparent in the hyperplastic lesions of HFD-fed mice at 12 weeks after feeding (Fig. 1c). A high frequency of Ki67-positive proliferating cells was observed in the hyperplastic and dysplastic stomach lesions of the HFD-fed mice, whereas these cells presented a defined proliferating zone in the CD-fed mice (Fig. 1e). These alterations rapidly progressed by 8 weeks of feeding (Fig. 2c). At 20 weeks of feeding, the folds of the glossy gastric mucosa were flat with a pale appearance, and polyp-like lesions were observed in the cardia and fundic regions of HFD-fed mice (arrows in Fig. 2a). At this point, the cell infiltration was complete, and the normal gastric glands were replaced by intestinal crypt-like structures at the cardial basal mucosa of HFD-fed mice (Fig. 2b). These results indicate that HFD-feeding alters gastric epithelial integrity even at the early phase of feeding and suggest that the occurrence of mucosal hyperplasia in the stomach was initiated by inflammation-independent events.

### Upregulation of intestinal markers in the gastric mucosa

**Fig. 3:**
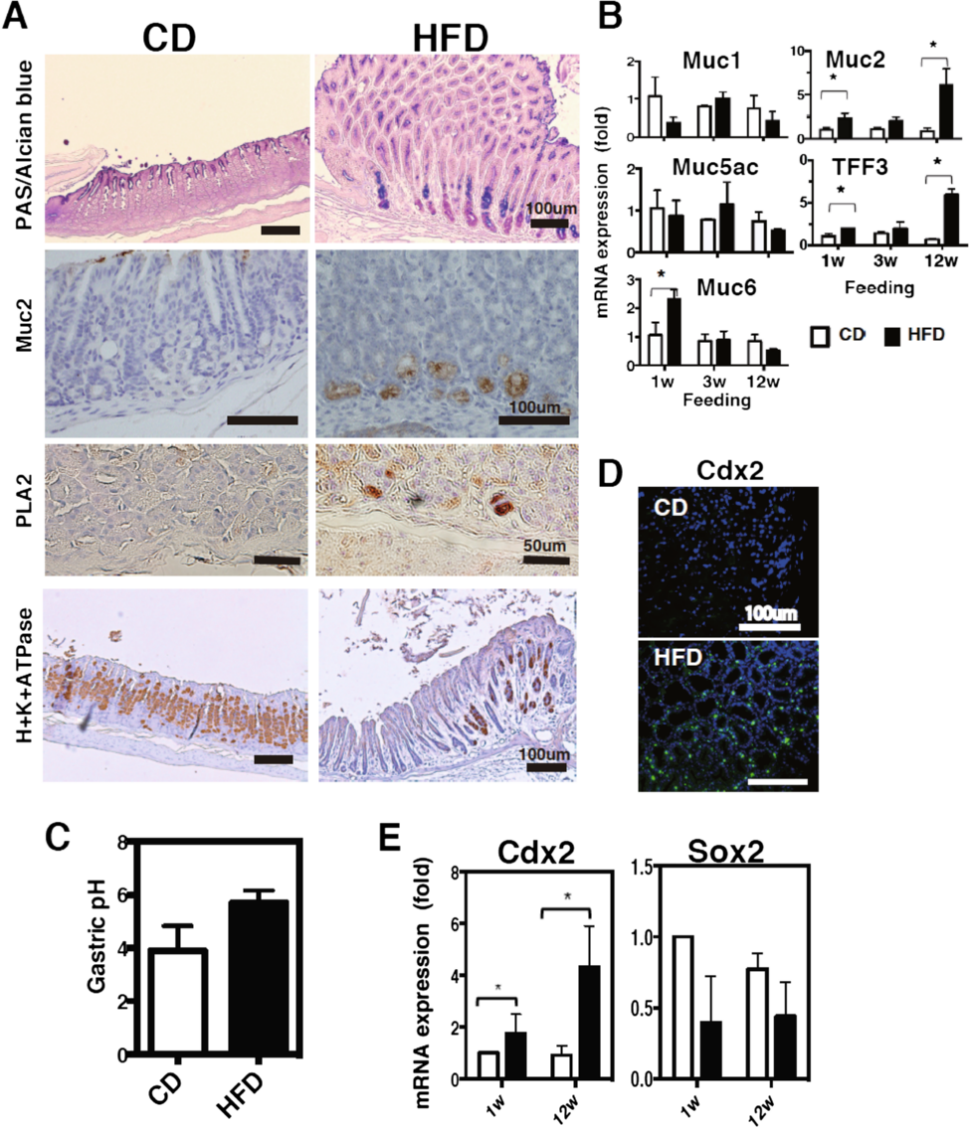
Alteration of intestinal characteristics in the gastric mucosa of HFD-fed mice. Gastric sections stained for PAS-Alcian blue, Muc2, PLA2, and H^+^K^+^ ATPase (**a**), and Cdx2 (**d**) in mice fed CD or HFD for 20 weeks. Gene expression of mucins (**b**), and *Cdx2* and *Sox2* (**e**) in the gastric mucosa of mice fed CD or HFD at 1 to 12 weeks after feeding. We utilized 5–10 mice in each analysis, and representative data are shown. **c** The gastric pH in the gastric lumen was measured according to the description provided in the Methods. Values represent the means ± SD of 5 mice. The results were analyzed by the Kruskal-Wallis test. * *p* < 0.05

We further examined whether the gastric mucosa in HFD-fed mice exhibited features of intestinal mucosa. We observed that the frequency of Alcian blue-stained goblet cells, which are intestinal mucus cells, increased as they spread across the gastric mucosa in HFD-fed mice (Fig. 3a). Muc2, an intestinal type of mucin, was ectopically expressed in the gastric mucosa only of HFD-fed mice (Fig. 3a), indicating that gastric mucin was converted to the intestinal type as has been observed in most human gastric carcinomas [24]. The results of gene expression analysis were consistent with the immunohistological findings. The mRNA expression of *Muc2* and *Tff3* (a peptide co-expressed with Muc2) increased, whereas that of the gastric-type mucins, *Muc1*, *Muc5ac*, and *Muc6*, remained unaltered or decreased in the stomachs of HFD-fed mice (Fig. 3b). The cardia of HFD-fed mice also showed ectopic expression of phospholipase A2 (PLA2), an intestinal Paneth cell marker (Fig. 3a). In contrast, the expression of H^+^K^+^-ATPase, a marker of parietal cells that secrete gastric acid, decreased with a concurrent elevation of gastric pH in the HFD-fed mice (Fig. 3a and 3c). In addition, the deregulation of transcription factor expression transposes into a metaplastic phenotype. Accordingly, the mRNA expression of *Cdx2*, an intestinal master transcription factor that is a marker of intestinal metaplasia, was higher in HFD-fed mice at 1 week (Fig. 3e). Furthermore, *Cdx2* mRNA expression was increased in the gastric mucosa of HFD-fed mice at 12 weeks in contrast to that observed in CD-fed mice, in which it was constant (Fig. 3d and 3e). Consistent with the ectopic *Cdx2* mRNA expression, the mRNA of *Sox2*, a transcription factor for stomach organogenesis [25], tended to be downregulated, indicating the development of intestinal metaplasia in the gastric mucosa in HFD-fed mice (Fig. 3e). Taken together, these results imply that HFD-feeding accelerates intestinal metaplasia.

### HFD feeding activates early leptin receptor signaling during gastric intestinal metaplasia

**Fig. 4:**
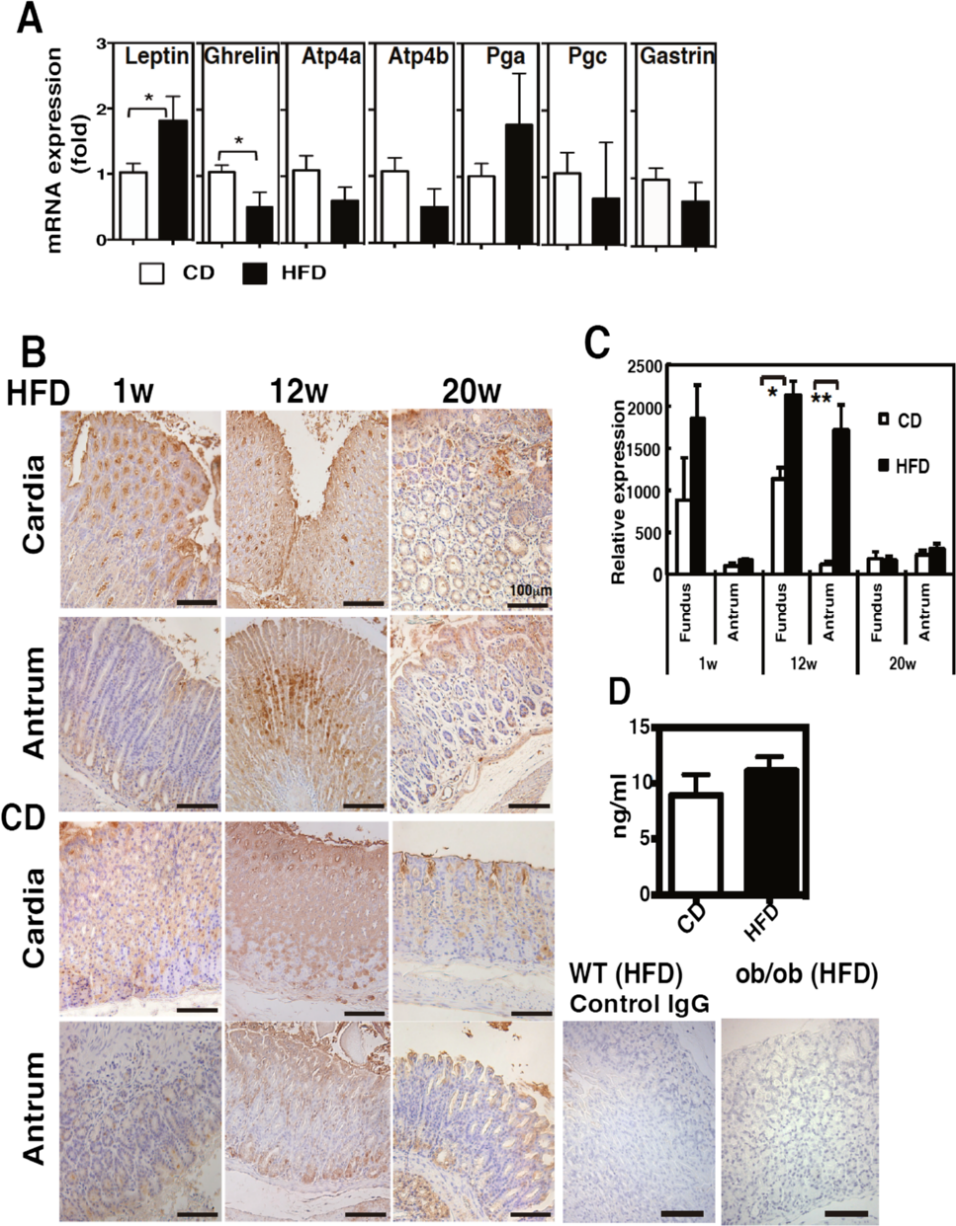
Enhancement of gastric leptin expression in the early period of HFD feeding. **a** Gene expression of gastric-specific molecules at 1 week after CD and HFD feeding. **b** Gastric sections stained for leptin in mice fed CD or HFD for 1, 12, and 20 weeks. **c** Quantification of leptin expression in (**b**). The data are presented as the relative expression at each time-point to the expression level in the antrum of CD-fed mice after 1 week. **d** Serum leptin concentration in mice fed CD and HFD for 1 week. The values represent the means ± SD of 5 mice. The results were analyzed by the Mann–Whitney *U* test. * *p* < 0.05, ** *p* < 0.01

**Fig. 5:**
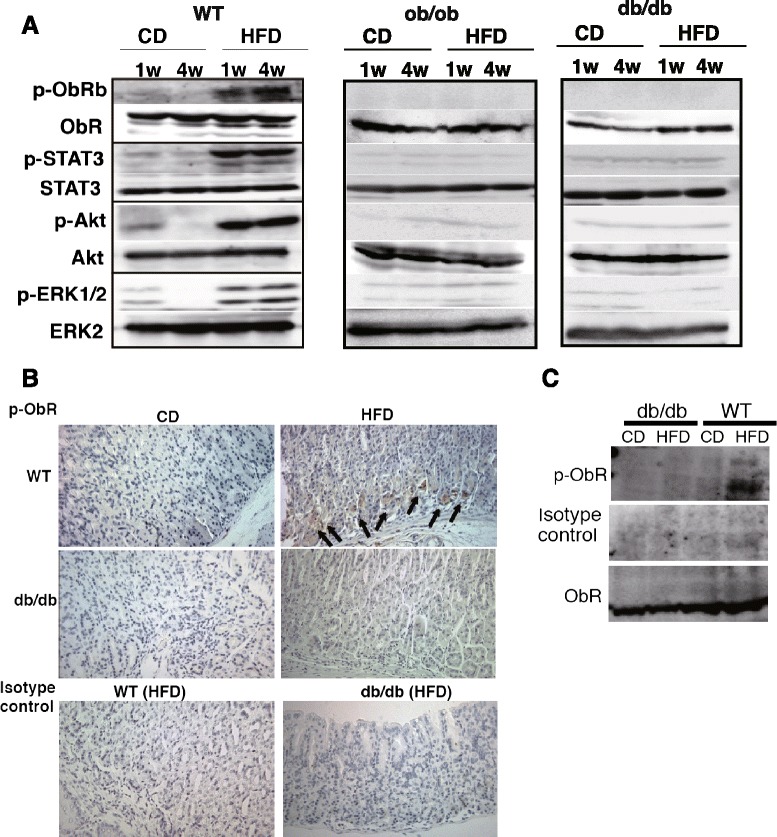
Upregulation of leptin receptor signaling in the gastric mucosa of HFD-fed WT mice. **a** Western blot analysis of the phosphorylation of ObRb, STAT3, Akt and ERK1/2 in the gastric mucosa of WT, *ob/ob* and *db/db* mice fed CD and HFD for 1 and 4 weeks. A total of 3 mice were used in each diet group per one experiment. Each experiment was repeated twice and representative data are shown. **b** Immunohistochemistry and (**c**) western blot analysis of phosphorylation of ObR in the gastric mucosa of *db/db* mice and WT mice fed CD or HFD for 3 weeks. We utilized 3 mice in each analysis, and representative data are shown. Arrows indicate phosphorylated ObR-positive cells

Because of the early morphological alterations observed in the gastric mucosa, we examined the gene expression of stomach-specific hormones, peptides, and enzymes 1 week after HFD feeding. Leptin mRNA expression in particular was significantly higher in the stomach of HFD-fed mice; in contrast, ghrelin expression was lower in HFD-fed mice (Fig. 4a). The expression of other genes such as *Atp4a*, *Atp4b*, *Pga*, and *Pgc*, which encode to H^+^K^+^-ATPase, pepsinogen A, and pepsinogen C, respectively, did not show significant differences between CD- and HFD-fed mice, although the expression of *Atp4a* and *Atp4b* tended to decrease (Fig. 4a). Normally, leptin is expressed in chief and parietal cells [26, 27]; however, HFD-fed mice exhibited strong leptin expression in the hyperplastic gastric epithelia (Fig. 4b and 4c). Even though the serum leptin concentration was similar between CD- and HFD-fed mice at 1 week after feeding (Fig. 4d), the leptin expression in the hyperplastic region of the stomach was consistent with the structural changes observed on H&E stained sections at 1 week (Fig. 1b). The leptin expression continued to increase to 12 weeks post HFD feeding, after which the expression approximately recovered to the level observed in CD-fed mice at 20 weeks (Fig. 4b and 4c). Concomitant with the high leptin expression at 1 week, phosphorylation of ObRb, STAT3, Akt, and ERK, which are molecules associated with leptin receptor signaling, was detected in the gastric mucosa of HFD-fed WT mice (Fig. 5a). These molecules remained activated at 4 weeks after feeding. In contrast, leptin-deficient *ob/ob* and ObR mutated *db/db* mice did not show phosphorylated ObRb, and only exhibited slightly activated STAT3, Akt, and ERK. In support of the validity of these findings, the isotype-control Ab did not react specifically and no expression of p-ObRb was detected in *db/db* mice (Fig. 5b and 5c).

**Fig. 6:**
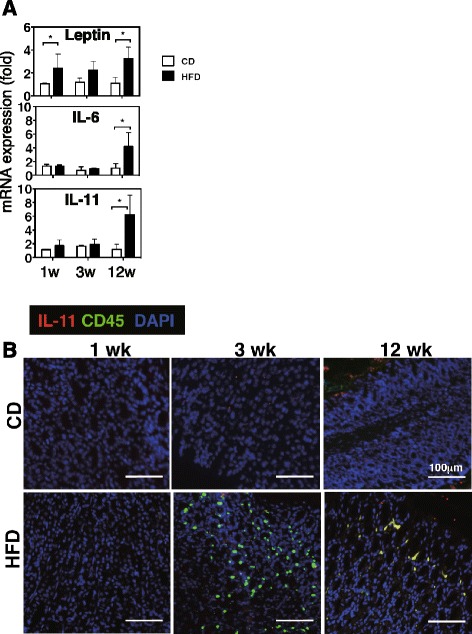
Delayed induction of proinflammatory cytokines in the gastric mucosa after HFD-feeding. **a** Gene expression of leptin, *Il6*, and *Il11* in the gastric mucosa of CD- and HFD-fed mice after 1, 3, and 12 weeks. The values represent the means ± SD of 4 mice. The results were analyzed by the Kruskal-Wallis test. * *p* < 0.05, *p* < 0.01. **b** Immunohistological staining of the stomach sections of the CD and HFD-fed mice with Abs against IL-11 and CD45

Chronic inflammation can trigger atrophic gastritis. IL-6 and IL-11, which are predominantly expressed in the stomach, modulate inflammatory responses during neoplastic progression [28, 29]. In particular, IL-11 expression increases in atrophic gastritis and in intestinal metaplasia of the fundic mucosa [30]. To identify potential initiators of intestinal metaplasia, we examined the expression of leptin, IL-6, and IL-11 in the gastric mucosa. Whereas leptin was expressed at 1 week after feeding, *Il6* and *Il11* mRNA levels did not increase in the gastric cardia of HFD-fed mice until 3 weeks after diet initiation (Fig. 6a). At 12 weeks, IL-11 expression accompanied an increased number of CD45^+^ infiltrated cells (Fig. 6a and 6b). These findings suggest that HFD-induced leptin expression and downstream activation precedes the induction of inflammatory cytokines during intestinal metaplasia.

### Lack of leptin signaling suppresses gastric intestinal metaplasia

**Fig. 7:**
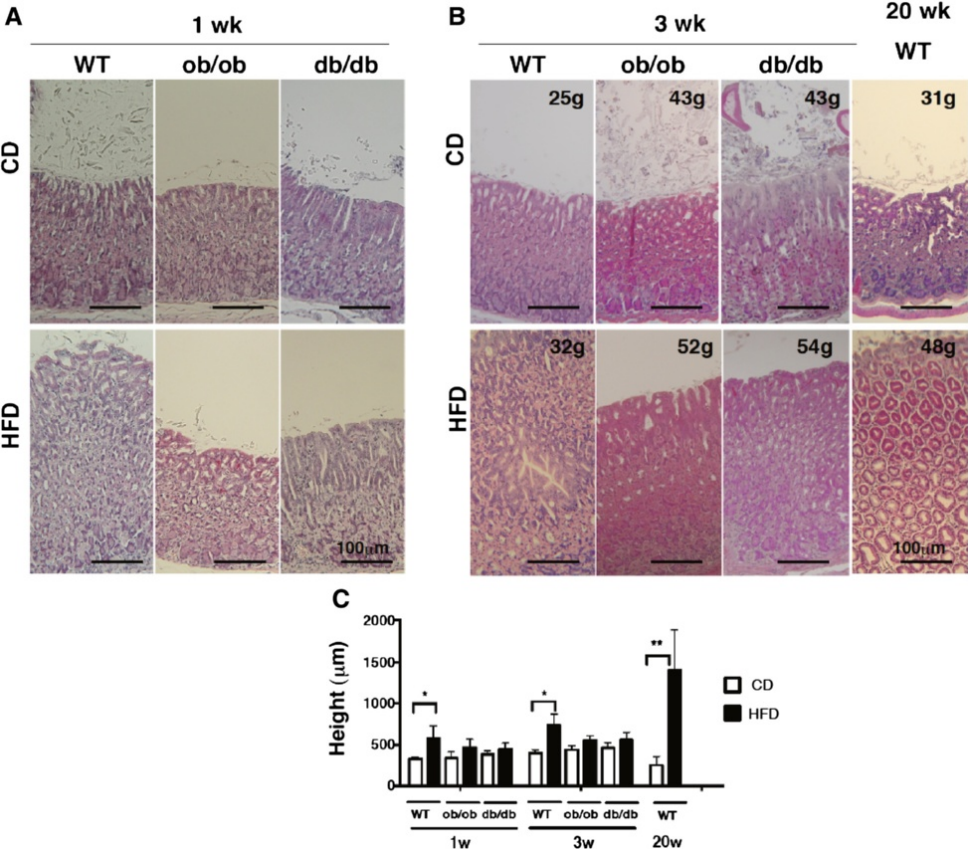
Suppressed alteration of gastric morphology in HFD-fed mice devoid of leptin signaling. Representative H&E-sections of the gastric mucosa from WT, *ob/ob* and *db/db* mice fed CD or HFD for 1 (**a**) and 3 weeks (**b**). Each value in the images indicates the body weight of the mouse from which the gastric mucosa was obtained and stained with H&E-stain. **c** Measurement of mucosal height in the gastric fundus of WT, *ob/ob* and *db/db* mice at 1 and 3 weeks and of WT mice at 20 weeks after feeding. The values represent the means ± SD of 4 mice. The results were analyzed by the Kruskal-Wallis test. * *p* < 0.05, ** *p* < 0.01

**Fig. 8:**
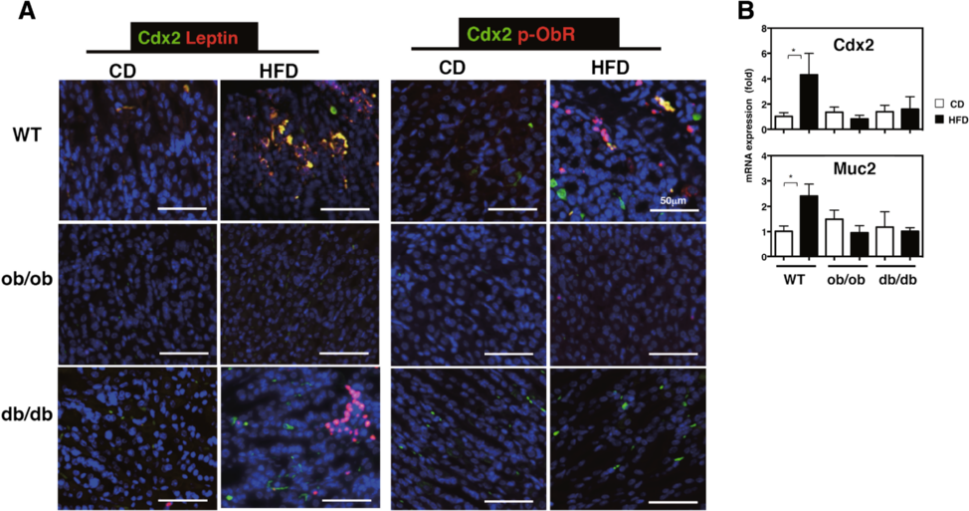
Downregulation of intestinal epithelial markers in mice devoid of leptin signaling. **a** Immunohistological staining of the stomach sections of the CD and HFD-fed mice with Abs against leptin, Cdx2, and p-ObRb. **b** Gene expression of *Cdx2* and *Muc2* in the gastric mucosa of mice fed CD or HFD for 1 week. The values represent the means ± SD of 4 mice. The results were analyzed by the Kruskal-Wallis test. *p < 0.01

HFD feeding activated leptin signaling, leading to intestinal metaplasia in WT mice. Conversely, the absence of leptin signaling should therefore suppress HFD-induced gastric pathology. To investigate the role of leptin signaling in the development of intestinal metaplasia, we examined gastric mucosal changes in leptin-deficient *ob/ob* mice and leptin receptor-mutated *db/db* mice. The *ob/ob* and *db/db* mice showed a higher body weight than did WT mice; however, no significant difference in body weights was observed between CD- and HFD-fed mice at 1 week after feeding (Additional file 3: Figure S1a). The *ob/ob* and *db/db* mice fed the CD for 1 week exhibited hyperinsulinemia, whereas only HFD-fed *ob/ob* mice showed increased insulin levels. HFD-fed *db/db* mice showed insulin levels similar to those of CD-fed *db/db* mice, because of their insulin resistance (Additional file 3: Figure S1b). The *db/db* mice also exhibited hyperleptinemia and hypergluconemia. However, the non-esterified fatty acid (NEFA) concentration did not vary among WT, *ob/ob*, and *db/db* mice fed with either CD or HFD (Additional file 3: Figure S1b). Histopathological analysis revealed that compared to the WT mice, *ob/ob* and *db/db* mice that were obese and insulin- or leptin- resistant showed few morphological changes such as hyperplasia of the gastric cardial mucosa at 1 week after initiation of HFD feeding (Fig. 7a). Mucosal height analysis was consistent with the histological findings (Fig. 7c). Furthermore, immunohistochemical analysis revealed increased colocalization of Cdx2 and leptin in the gastric mucosa of WT mice fed the HFD for 1 week (Fig. 8a), whereas *db/db* mice did not show Cdx2 expression in leptin-positive cells. In contrast, *ob/ob* mice showed little Cdx2 and no leptin expression. Similarly, co-localization of phosphorylated ObRb and Cdx2 was detected in HFD-fed WT mice, but not in HFD-fed *ob/ob* or *db/db* mice, which showed only some phosphorylation of ObRb or Cdx2, respectively. CD-fed ob/ob and db/db mice showed a drastic increase in body weight but only slight hyperplasia in the gastric mucosa compared to WT mice at 3 weeks (Fig. 7b and 7c). Despite having higher body weights, *ob/ob* and *db/db* mice fed the HFD for 3 weeks exhibited less hyperplasia than did WT mice fed the HFD for 20 weeks. After 20 weeks of HFD feeding, the WT mice presented an irregular and fused structure in the cardia (Fig. 7b). HFD feeding did not increase the expression of Cdx2 and Muc2 in the gastric mucosa of *ob/ob* and *db/db* mice (Fig. 8b). These results suggest that leptin signaling in the stomach is an important factor that leads to metaplastic pathology in obesity-related gastritis.

## Discussion

This study presents the first evidence supporting the theory that HFD-induced enhanced leptin receptor signaling in the gastric mucosa causes atrophic gastritis in a murine model. We found that HFD feeding triggers leptin production and the activation of leptin-ObR signaling in the gastric mucosa, promoting atrophic gastritis and intestinal metaplasia. Given the higher frequency of gastritis in obese patients [31, 32], our results substantiate the role of leptin receptor signaling in obesity-induced atrophic gastritis. *H. pylori* infection is considered a major cause of chronic gastritis and GC [33, 34]; however, very few *H. pylori*-infected patients develop GC [35]. Furthermore, the frequency of gastritis is significantly higher in morbidly obese patients, whereas the prevalence of *H. pylori* infection is similar in obese and non-obese individuals [2]. Thus, obesity is an important factor accounting for the prevalence of histologic gastritis and the transformation of gastric mucosal cells in addition to *H. pylori* infection.

In addition to adipose tissue, the placenta [36] and mammary glands [37, 38] also produce leptin, which supports trophoblast cell proliferation to regulate fetal growth and development [39, 40] and the development of the mammary gland, respectively. In those tissues, leptin is produced in both normal and malignant cells, and its overexpression has been demonstrated in neoplastic cells [36, 41]. Elevated expression of leptin and ObRb is associated with faster tumor recurrence and mortality in human grade-III invasive breast tumors [42, 43]. Tumor cells derived from MMTV-Wnt1 mice, a widely used model of mammary tumors, show decreased growth in *ob/ob* mice compared with cells from diet-induced obese mice that have functional leptin signaling [44]. Our data show that *ob/ob* and *db/db* mice show no activation of the ObR, a marked decrease in the activation of STAT3, Akt, and ERK (Fig. 5a), and exhibit less progression of gastric hyperplasia even though these mice are more obese than HFD-fed WT mice (Fig. 7). Furthermore, *ob/ob* and *db/db* mice do not spontaneously develop gastric tumors although these mice exhibit extraordinary obesity. Thus, the results of our study together with those of the previous reports imply that leptin signaling is critically involved in the control of epithelium development in a variety of tissues. Although the significance of leptin expression and its signaling in the stomach remains uncertain, considering the constitutive expression of leptin and ObRb in the stomach [45, 46], leptin might be necessary for the maintenance of gastric mucosal homeostasis.

Leptin is considered to represent a key player in obesity-associated gastrointestinal malignancies because of its roles in angiogenesis, apoptosis, cell proliferation, and cell migration, which support the milieu of tumor development and progression [17]. Recently, leptin was shown to contribute to mucin production and the formation of gastrointestinal neoplasms by modulating mTOR-, STAT3-, and ERK-dependent pathways [47] in addition to PKC-, PI3K-, and MAPK-dependent pathways [48, 49]. The mechanisms underlying the induction of gastric atrophy are not defined; however, transcription factors responsible for cell differentiation have been reported to be potently involved in gastric tumorigenesis. Transcription factors such as Cdx2 and Sox2 are critical for cell self-renewal and the maintenance of cells in appropriate proportions [50]. Sox2 is a primary regulator of gastric differentiation that maintains gastric mucosal features and regulates the expression of gastric mucus genes, such as *Muc5ac* [51], and it is downregulated during the development of gastric adenocarcinoma [52, 53]. In contrast, Cdx2 is essential for intestinal epithelium differentiation to regulate the expression of Muc2 [54, 55] and Tff3 [56], and it is upregulated during the progression of gastric tumors [57]. Recently, the binding of phosphorylated STAT3 to the Cdx2 promoter region was shown to upregulate Cdx2 but downregulate Sox2 in two gastric cell lines, MKN45 and NUGC4 [58]. In this study, we showed that HFD feeding induces Cdx2 and Muc2 expression associated with increased leptin levels (Figs. 3 and 8), suggesting the possibility that leptin acts to suppress cellular protection against gastric mucosal malignancies in the early stage of atrophic gastritis.

Atrophic gastritis is characterized by an alteration of the differentiation of gastric mucosal cells such as the loss of parietal and chief cells and replacement of the destroyed glandular structure with a diffuse intestinal mucous metaplasia [59]. Variations in the pathology of atrophic gastritis have been reported to some degree in genetically engineered mice such as Atp4a^−/−^ and gastrin^−/−^ mice, which are used as models of parietal and glandular cell loss. However, Atp4a^−/−^ mice exhibit the same expression levels of Muc2 and Tff3 in the gastric mucosa as control Atp4a^+/+^ mice, indicating incomplete intestinalization of the gastric mucosa in these mice [60]. Gastrin is secreted from G-cells to stimulate acid secretion and cell proliferation of the fundic mucosa [61]. Gastrin^−/−^ mice develop antral gastric neoplasia with increased levels of Muc2 and villin (an intestinal-specific actin bundling protein) in the gastric mucosa in the presence of gastric inflammation but not in the fundus [62, 63]. As proinflammatory cytokines involved in atrophic gastritis, IL-6 and IL-11 cause an inflammatory response in the stomach and play important roles in angiogenesis and cell proliferation during the progression of neoplasia [28, 29]; thus, they are used as biomarkers of aggressive tumors [64]. In particular, IL-11, which is mainly expressed in the fundic mucosa, might play a pivotal role in atrophic gastritis and the progression of GC [30, 65]. Chronic treatment of WT mice with recombinant IL-11 induces atrophy in the gastric fundus, including the cardia, with reduced parietal cells and highly phosphorylated STAT3, but no features of intestinalization of the gastric mucosa such as the appearance of goblet cells, Muc2, and Cdx2. These reports raise the question of which molecules are directly involved in the atrophy of the gastric mucosa associated with metaplasia. In our study, the increase of gastric leptin expression is observed during the early period after the initiation of HFD feeding and its subsequent reduction is consistent with the loss of normal parietal and chief cells, which constitutively produce leptin and ObR [26, 27], in the gastric mucosa of HFD-fed mice in accordance with the development of intestinal metaplasia after 20 weeks of HFD feeding (Figs. 1, 2 and 3). Our results might therefore directly indicate the involvement of leptin receptor signaling in the initiation of obese-associated gastric atrophy.

The involvement of gastric leptin in diet-induced obesity-associated gastric atrophy has not previously been investigated, although the elevation of serum leptin contributes to tumorigenesis in the stomach of obese mice infected with *H. felis* [66]. In the current study, we showed rapid (1 week) mucosal morphological changes in the gastric cardia (Fig. 1b) in accordance with high expression of gastric leptin at this time (Fig. 4d). Furthermore, prior to the production of IL-11, leptin expression in the cardial gastric mucosa increases, concurrent with the hyperplastic lesions observed in the early period after HFD feeding (Fig. 4b and 6). The localization of leptin-producing cells within cardial mucosal lesions occurs during the early stage of atrophy in HFD-fed mice. Furthermore, the increased leptin levels and activation of ObRb resulted in increased levels of phosphorylated STAT3 in the HFD-fed mice (Fig. 5a). Notably, because *ob/ob* and *db/db* mice do not transduce leptin receptor signaling and do not show morphological alterations such as loss of glandular structure (Fig. 5a and 7), the elevation of gastric leptin by HFD feeding might initiate a reduction of glandular structure by diffuse metaplasia, i.e., a loss of parietal and chief cells. Since most parietal and chief cells residing in the fundic mucosa of the stomach express leptin and ObRb [26, 45, 46], it would be interesting to investigate how these cells respond to food-based stimulants such as fatty acids in the HFD.

In summary, we demonstrated the development of atrophic gastritis using a murine model of diet-induced obesity with an enhanced leptin-ObRb signaling pathway. The expression and unique localization of the leptin-ObR signaling pathway predominantly indicates a role in the early phase of human GC. The significance of this study lies in the potential and invaluable use of leptin and ObR as biomarkers or as new therapeutic targets for the diagnosis and treatment of atrophic gastritis.

## Conclusion

In this study, we show that diet-induced obese WT mice exhibit overexpression of leptin and activation of leptin receptor signaling in the gastric mucosa, leading to atrophic gastritis with intestinal metaplasia of the gastric mucosa. These pathological features are less severe in *ob/ob* and *db/db* mice, which lack leptin receptor signaling, than in the WT mice. Therefore, leptin receptor signaling in the stomach is a critical checkpoint for the onset of gastric neoplasia, and preventive therapeutics targeting gastric leptin receptor signaling might be developed against GC.

## Additional files

Additional file 1: Table S1. Antibody list: Antibodies used for western blotting and immunohistochemistry. (DOCX 127 kb) Additional file 2: Table S2. Primers used for quantitative PCR. (DOCX 100 kb) Additional file 3: Figure S1. Alteration of index associated with obesity. Body weight (A), insulin, leptin, glucose and NEFA in sera (B) in CD- and HFD-fed WT, ob/ob and db/db mice were measured 1 week after feeding. Results were analyzed by the Kruskal Wallis test. **p* < 0.01. (EPS 1473 kb)

## Abbreviations

CD: Control diet
DTT: Dithiothreitol
EDTA: Ethylenediaminetetraacetic acid
ELISA: Enzyme-linked immunosorbent assay
GC: Gastric carcinoma
HFD: High fat diet
LMD: Laser-capture microdissection
NEFA: Non-esterified fatty acid
WT: Wild-type
