# Supplementary material for: Leptin receptor signaling is required for high-fat diet-induced atrophic gastritis in mice
Source: Nutr Metab (Lond). 2016 Feb 2;13:7. doi: 10.1186/s12986-016-0066-1 (PMC4736478; doi:10.1186/s12986-016-0066-1)
Supplement: Additional file 1: Table S1. — Antibody list: Antibodies used for western blotting and immunohistochemistry. (DOCX 127 kb) [file 12986_2016_66_MOESM1_ESM.docx]

Supplemental Table 1. Antibody list: Antibodies used for western blotting and immunohistochemistry

| **Peptide/protein target** | **Antigen sequence** | **Antibody name** | **Manufacturer catalog No.** | **Species raised in; monoclonal or polyclonal** | **Dilution used** |
| --- | --- | --- | --- | --- | --- |
| p-STAT3 | Phosphopeptide corresponding to residues surrounding Tyr705 of mouse Stat3 | Phospho-Stat3 (Tyr705) (D3A7) | Cell Signaling (#9145) | Rabbit polyclonal | 1:1000 (WB) 1: 200 (IHC) |
| STAT3 | Peptide mapping at the C-terminus of Stat3 of mouse origin | Stat3 (C-20) | Santa Cruz Biotech (sc-482) | Rabbit polyclonal | 1: 500 (WB) |
| p-ObR | Amino acid sequence containing phosphorylated Tyr1138 of Ob-R of mouse origin | p-Ob-R (Tyr1138) | Santa Cruz Biotech (sc-16421) | Goat polyclonal | 1: 200 (WB)  1: 50 (IHC) |
| ObR | Peptide mapping at the N-terminal extracellular domain of Ob of mouse origin | Ob-R (I-17) | Santa Cruz Biotech (sc-33982) | Goat polyclonal | 1: 200 (WB) |
| p-Akt | Phosphopeptide corresponding to residues around Ser473 of human Akt. | Phospho-Akt (Ser473) (D9E) | Cell Signaling (#4060) | Rabbit monoclonal | 1:1000 (WB) |
| Akt | Peptide corresponding to the C-terminal sequence of mouse Akt. | Akt antibody | Cell signaling (#9272) | Rabbit polyclonal | 1:1000 (WB) |
| p-ERK  1/2 | Phosphopeptide corresponding to residues surrounding Thr202/Tyr204 of human p44 MAPK | Phospho-p44/42 MAPK (Erk1/2)(Thr202/Tyr204) (E10) | Cell Signaling (#9106) | Rabbit monoclonal | 1:1000 (WB) |
| ERK2 | peptide mapping at the C-terminus of ERK2 of rat origin | ERK2 (C-14) | Santa Cruz Biotech (sc-154) | Rabbit polyclonal | 1: 200 (WB) |
| Ki67 | Synthetic peptide conjugated to KLH derived from within residues 1200 - 1300 of Human Ki67 | Rabbit polyclonal to Ki67 | Abcam Inc., (ab15580) | Rabbit polyclonal | 1: 200 (IHC) |
| Na+/K+ ATPase | Amino acids residues 1-13 or 15-28 located on the cytoplasmic side of the beta-subunit | Hydrogen potasium ATPase beta (2G11) | Abcam Inc., (ab2866) | Mouse monoclonal | 1:2500  (IHC) |
| Muc2 | Amino acids 4880-5179 mapping at the C-terminus of Mucin 2 of human origin. | Mucin 2 (H-300) | Santa Cruz Biotech (sc-15334 ) | Rabbit polyclonal | 1: 100 (IHC) |
| IL-11 | Amino acids 31-199 of IL-11 of human origin | IL-11 (H-169) | Santa Cruz Biotech (sc-7924 ) | Rabbit polyclonal | 1: 50 (IHC) |
| PLA2 | Synthetic non-phospho- peptide derived from human Phospholipase A2 around the phosphorylation site of serine 505 | Anti-Phospho-  lipase A2 antibody | Abcam Inc., (ab58375) | Rabbit polyclonal | 1: 1000 (IHC) |
| Cdx2 | 180 amino acids from the N-terminal region of Cdx2 of human origin | Cdx2 (AMT28) | Santa Cruz Biotech (sc- 56818) | Mouse monoclonal | 1: 50 (IHC) |
| Leptin | Synthetic peptide: SRNVIQISNDLENLRD, corresponding to amino acids 91-106 of Human Leptin | Anti-Leptin antibody | Abcam Inc., (ab16227) | Rabbit polyclonal | 1: 400 (IHC) |
| CD45 | 30-F11 | Purified Rat Anti-Mouse CD45 | BD Pharmingen | Rat monoclonal | 1: 50 (IHC) |
| Goat IgG | Affinity purified isotype control immunoglobulin  from goat. | Normal goat IgG | Santa Cruz Biotech (sc- 2028) | Goat Immunogloblin | 1: 200 (WB)  1: 50 (IHC) |
| Rabbit IgG | Affinity purified isotype control immunoglobulin  from rabbit | Rabbit IgG, polyclonal-isotype control | Abcam Inc. (ab27478) | Rabbit Immunogloblin | 1: 400  (IHC) |
