# Supplementary material for: Leptin receptor signaling is required for high-fat diet-induced atrophic gastritis in mice
Source: Nutr Metab (Lond). 2016 Feb 2;13:7. doi: 10.1186/s12986-016-0066-1 (PMC4736478; doi:10.1186/s12986-016-0066-1)
Supplement: Additional file 2: Table S2. — Primers used for quantitative PCR. (DOCX 100 kb) [file 12986_2016_66_MOESM2_ESM.docx]

Supplemental Table 2: Primers used for quantitative PCR

| Name |  | Primer sequences | Accession Number |
| --- | --- | --- | --- |
| *Muc1* | Sense | 5′-aatggcactcagccttcagt-3′ | NM_013605.2 |
|  | Anti-sense | 5′-gaaggagaccccaacagaca-3′ |  |
| *Muc2* | Sense | 5′-acaaaaaccccagcaacaag-3′ | NM_023566.3 |
|  | Anti-sense | 5′-gagcaagggactctggtctg-3′ |  |
| *Muc5ac* | Sense | 5′-gcaactggaccaagtggttt-3′ | NM_010844.1 |
|  | Anti-sense | 5′- tgacccagatcctccatctc-3′ |  |
| *Muc6* | Sense | 5′-tgcatgctcaatggtatggt-3′ | NM_181729.2 |
|  | Anti-sense | 5′- tgtgggctctggagaagagt-3′ |  |
| *Tff3* | Sense | 5′-cagattacgttggcctgtctcc-3′ | NM_011575.2 |
|  | Anti-sense | 5′- atgcttgctacccttggccac-3′ |  |
| *Leptin* | Sense | 5′-tgacaccaaaaccctcatca-3′ | NM_008493.3 |
|  | Anti-sense | 5′- tcattggctatctgcagcac-3′ |  |
| *Ghrelin* | Sense | 5′- tctgcagtttgctgctactca-3′ | NM_001286404.1 |
|  | Anti-sense | 5′-cctctttgacctcttcccaga-3′ |  |
| *Atp4a* | Sense | 5′-gtctggagggaacagctcag-3′ | NM_001290627.1 |
|  | Anti-sense | 5′ -taccacaatggccatgaaga-3′ |  |
| *Atp4b* | Sense | 5′- ccttgagaccggacgtgt at-3′ | NM_009724.2 |
|  | Anti-sense | 5′-ggcctgagcagttttgtagc-3′ |  |
| *Gastrin* | Sense | 5′-accaatgaggacctggaaca-3′ | NM_010257.3 |
|  | Anti-sense | 5′-atccatccgtaggcctcttc-3′ |  |
| *Pga* | Sense | 5′-cctcaggagttcagggttgt-3′ | NM_021453.4 |
|  | Anti-sense | 5′-tccccaatcctgacagtgtc-3′ |  |
| *Pgc* | Sense | 5′-cctgtgggtgtcttctgtct-3′ | NM_025973.3 |
|  | Anti-sense | 5′-tagggacctggatgctttgg-3′ |  |
| *Cdx2* | Sense | 5′- tctccgagaggcaggttaaa -3′ | NM_007673.3 |
|  | Anti-sense | 5′- gcaaggaggtcacaggactc -3′ |  |
| *Sox2* | Sense | 5′-cacaactcggagatcagcaa-3′ | NM_011443.3 |
|  | Anti-sense | 5′-ctccgggaagcgtgtactta-3′ |  |
| *18S* | Sense | 5′-gtaacccgttgaaccccatt-3′ | NM_003278.3 |
|  | Anti-sense | 5′-ccatccaatcggtagtagcg-3′ |  |
